# A Scoping Review of the Influence of Mindfulness on Men’s Sexual Activity

**DOI:** 10.3390/ijerph20043739

**Published:** 2023-02-20

**Authors:** María Fernanda Valderrama Rodríguez, Laura C. Sánchez-Sánchez, José Manuel García-Montes, Cristina Petisco-Rodríguez

**Affiliations:** 1 Department of Psychology, University of Almería, Carretera Sacramento, S/N, La Cañada de San Urbano, 04120 Almería, Spain; 2 Department of Personality, Evaluation and Psychological Treatment, Faculty of Psychology, University of Granada, Campus Cartuja, SN, 180071 Granada, Spain; 3 Faculty of Education, Pontifical University of Salamanca, Calle Henry Collet, 52-70, 37007 Salamanca, Spain

**Keywords:** mindfulness, male sexuality, sexual dysfunctions, sexual desire, erectile dysfunction, sexual satisfaction

## Abstract

Mindfulness practice and mindfulness-based interventions are widely known, especially for women’s sexuality. However, it is currently unknown how this practice affects the experience of male sexuality, possibly due to the existence of pharmacological treatments that are usually the first choice of treatment for men. The objectives of this study are to explore the influence of mindfulness on different components of men’s sexuality from a scoping review of relevant scientific articles existing in the literature. A literature search from 2010 to 2022 was carried out in the electronic databases MEDLINE, Embase, PsycINFO, Web of Science, Scopus, PubMed, Dialnet, SciELO Citation Index, and Redalyc. Out of the 238 studies, 12 that met the defined selection criteria were selected. The analysis of these studies seems to indicate that the practice of mindfulness favours different variables of male sexuality, such as satisfaction and sexual functioning or genital self-image. Mindfulness-based interventions represent a valuable and promising contribution. No adverse effects were detected from the review of scientific articles considered in this work. Nevertheless, more randomized studies with active control groups are necessary to establish the benefits of mindfulness-based interventions in sex therapy for men.

## 1. Introduction

Sexual health is fundamental to the overall health and well-being of individuals, couples, and families, and to the social and economic development of communities and countries. Sexual health, when viewed affirmatively, requires a positive and respectful approach to sexuality and sexual relationships, as well as the possibility of having pleasurable and safe sexual experiences, free of coercion, discrimination, and violence [1]. This view highlights the diversity of the expression of each person’s sexuality in thinking, feeling, and acting; that is, with the diversity provided by each person’s learning history and current behaviour.

Although, from the perspective of contextual therapies, sexual problems are understood according to a transdiagnosis, the fact is that the databases continue to use diagnoses according to classifications of mental disorders. Currently, in the DSM-V manual, the following sexual dysfunctions are included: delayed ejaculation, erectile disorder, hypoactive sexual desire disorder in men, and premature ejaculation. Delayed ejaculation occurs in 1% to 4% of the world’s population [2,3,4]. Erectile dysfunction is expected to account for nearly 322 million cases by 2025 [5]. The prevalence of hypoactive sexual desire disorder in men worldwide is unknown; however, 14.4% of men in Portugal, Croatia, and Norway reported a distressing lack of sexual desire lasting at least 2 months [6]. In the United States, the self-reported prevalence in a sample of men aged 40-80 years was 4.8%. Whereas 4.8% reported occasional lack of sexual desire, only 3.3% reported frequent lack of sexual desire [7]. Finally, premature ejaculation currently affects 20-30% of the world’s population [8].

There is ample scientific evidence on the various factors that may increase the risk of male sexual dysfunction, such as inadequate or absent sex education, life events, relationships, mental health, and lifestyle [9]. Lifestyle factors include alcohol consumption, psychoactive substances, physical inactivity, and diet [10,11,12]. This study considers all of these factors assessed within male sexuality and their relationship to mindfulness practice.

The practice of mindfulness dates back more than 2500 years; however, Jon Kabat-Zinn is the author who more recently, together with the University of Massachusetts, integrated the meditative practice from Buddhism in the West in order to make it a tool of intervention in patients with chronic pain. This program was called Mindfulness Based Stress Reduction (MBSR) [13]. As the program spread and became the object of multiple investigations, it has been possible to detail the benefits that emerged after its practice, which were limited to not only stress reduction or pain management, but also neurological changes and an increase in the subjective perception of the level of well-being [14].

The mindfulness concept can be understood as full consciousness, coming from the word Sati, from the Pali language [15]. Mindfulness “refers to the ability to become aware of the present. Practicing mindfulness makes individuals develop their awareness of either sensation, thought or activity (internal or external), without judgement and with radical acceptance” (p. 1) [16]. It can be understood not only as a state but also as a dispositional tendency or a stable trait [17].

Research indicates that the habitual practice of mindfulness tends to develop in people a series of capacities and characteristics [18]. Jon Kabat-Zinn raises some essential components within the practice of mindfulness, starting with no judging, described as living the continuous present without launching value judgements (neither positive nor negative) or assessments, being impartial, and being aware of internal or external stimulus. Attention must be focused on and occupied with the immediate experience, focusing on one aspect at a time. See everything as if it were the first time it was being observed, as if it were unknown, moving away from the previous experience to get closer to the current experience. Mindfulness invites us, therefore, to recognize the present internal or external experience as it appears. It involves understanding what is or is not happening, knowing that thoughts, feelings, actions, and beliefs are just that: thoughts, feelings, actions, and beliefs that occur in the present moment [19]. It also favours the cultivation of patience, which is necessary to have the confidence that things develop in their own time [20].

As a result, techniques related to mindfulness can be implemented and developed within sexual therapy, where tools are provided through theoretical information and practical exercises in and between sessions. The introduction of the practice of mindfulness in sexual therapy focuses on the fact that sexual dysfunctions and sexual problems are largely related to distractions, judgement, anxiety, inhibitions, self-criticism regarding performance, and lack of attention to sexual stimuli [21,22,23]. This results in self-demands that point more towards quantity, genitality, and social stereotypes than to quality, feeling, enjoyment, and eroticism.

The use of mindfulness in sexual therapy not only aims to solve a problem or disorder but, in congruence with its objective, mindfulness improves the quality of life of those who practice it. This implies that their learning can be aimed at improving attention and concentration, awakening all the senses, enjoyment, tolerance to discomfort, and experiencing the present without judgement expectations, beliefs, and feelings of guilt. Another advantage of introducing the practice of mindfulness in sexual therapy is that it can be done in a couple or alone; that is, a single person experiencing sexual preoccupations outside the context of a relationship may seek sexual therapy, and the outcome of the practice will be effective (see Stephenson [24] for a review). There is scarce research and intervention to evaluate the associations of mindfulness training on couples’ romantic and sexual well-being, but the results of the study by Leavitt et al. [25] showed that couples have greater awareness and ability to not judge themselves or their partner, reporting increased satisfaction with the relationship and their sexual experience.

Studies that have been carried out relating to the practice of mindfulness and sexuality in men have revealed its effectiveness in sexual desire, the level of performance anxiety, sexual satisfaction and fantasies, and the use of pornography, among other variables [26,27,28]. Studies related to male sexual dysfunction have focused on the effect of mindfulness on reducing performance anxiety, thought fusion, and sexual desire, with the understanding that mindfulness practice may act as a mediator between anxiety and sexual desire [26,29,30,31]. Likewise, the effect of mindfulness-based interventions in reducing anxiety in men diagnosed with erectile dysfunction has been analysed, as men focus their attention on the sexual stimulation received rather than on distraction or emotional avoidance [32,33,34,35,36].

The effects of various mindfulness-based intervention protocols on women have been extensively evaluated. Women’s sexuality differs from men’s and relies much more on psychological than physiological factors [37,38]. In the study carried out by Silverstein et al. [39], women who underwent mindfulness meditation training improved their ability to detect their own physiological responses to sexual stimuli, and this was associated with improvements in attention, self-judgement, and clinical symptoms, which are known psychological barriers to healthy sexual functioning. However, the effects of these protocols on the experience of men’s sexuality are still largely unknown. This scientific knowledge could provide a novel perspective from which to clinically intervene in different sexual problems and, in turn, enable men to benefit from the effects of this practice, even when they are not part of a clinically relevant population. This would imply a shift towards a biopsychosocial framework for the treatment of male sexual dysfunction. In fact, mindfulness has been used and studied with women because there have been no other options, such as those presented for men. However, mindfulness can be effective for some sexual problems faced by men and does not result in the additional problems that some medications bring. Health professionals related to sexuality might consider incorporating mindfulness to address the psychosocial and psychosexual components of dysfunction [36].

The objectives of this study are, therefore, to explore the evaluation of the practice of mindfulness in sexual activity in men and to identify the different sex therapy interventions based on mindfulness in men, from the scoping review of relevant existing works in the scientific literature following the PRISMA method [40,41].

## 2. Materials and Methods

### 2.1. Search Strategy

A bibliographic search was carried out in the electronic databases MEDLINE, Embase, PsycINFO, Web of Science, Scopus, PubMed, Dialnet, SciELO Citation Index, and Redalyc. Studies from 2010 to June 2022 were analysed, as no scientific articles related to the evaluation of mindfulness practice in men were found before this period. The search was performed using the words “mindfulness and male sexuality”, “mindfulness and men sexuality”, “MBSR and male sexuality”, and “MBCT and male sexuality” in the databases in English, and “mindfulness y sexualidad masculina”, “mindfulness y sexualidad y hombres”, “MBSR y sexualidad masculina”, and “MBCT y sexualidad masculina” in Spanish databases. The review included original studies regardless of their methodology, the sample size, and the presence of a control group.

### 2.2. Selection Process

Descriptive, correlational, experimental, and quasi-experimental studies were considered, where an intervention in sexual therapy based on mindfulness was carried out. Studies on the effect of mindfulness practice on some variables of sexual activity in men were also taken into account. Articles highlighting different aspects of male sexuality from a biopsychosocial perspective were included, such as biological (sexual functioning, hormones, genetics), psychological (depression, anxiety, impulsivity, degree of aggressiveness, body image, degree of compassion, subjective sexual satisfaction), and social (relationship, alcohol consumption, sexuality awareness).

The inclusion criteria were as follows:

- Inclusion criteria 1 (I1): publication period between 2010 and 2022
- Inclusion criteria 2 (I2): the document is a scientific article
- Inclusion criterion 3 (I3): adolescents and adults (≥14 years old)
- Inclusion criteria 4 (I4): in English or Spanish

The exclusion criteria were as follows:

- Exclusion criterion 1 (E1): the full text is not available
- Exclusion criterion 2 (E2): studies where statistics related to men were not presented, even when they had been included as a sample in the study
- Exclusion criteria 3 (E3): does not address the selected topic (mindfulness in male sexuality)
- Exclusion criterion 4 (E4): bibliographic review studies or systematic reviews

The title, abstract, and keywords of each scientific article were reviewed to determine possible eligibility. If these data were not sufficient to determine whether they met the inclusion criteria, the full article was downloaded and reviewed. The selection of the studies was carried out by two reviewers (MFVR and LCSS), with a third (JMGM) if there were any disagreements between them. Studies that met the inclusion criteria were selected and their analyses were subsequently performed. The reviewers assessed the levels of quality and evidence of the studies that were included to determine their scientific value. The Jadad scale [42] was used for randomized controlled trials. This scale is scored from 0 to 5, and the higher the score, the higher the quality of the study. It assesses whether studies are randomized, whether they are double-blinded, and whether they describe loss to follow-up. For non-randomized controlled trials, the Estabrooks scale [43] was used. This scale ranges from 0 to 28 points and assesses six categories: (a) design and randomisation, (b) recruitment (inclusion and exclusion criteria), (c) loss control, (d) description of the intervention, (e) statistical analyses and conclusions, and (f) outcome measures ((see Results section)).

### 2.3. Data Collection Process

The following data were collected in a spreadsheet: author/s, year of publication, country, number of participants, method (type of intervention), methodological design, and results. For the qualitative analysis, NVIVO version 11 software was used to organize and explore the information in the scientific articles selected. This software, among other functions, analyses the content of the documents and searches for the most frequent words coinciding in them, which is indicative of the relative importance attributed to these words in these studies.

## 3. Results

**Figure 1:**
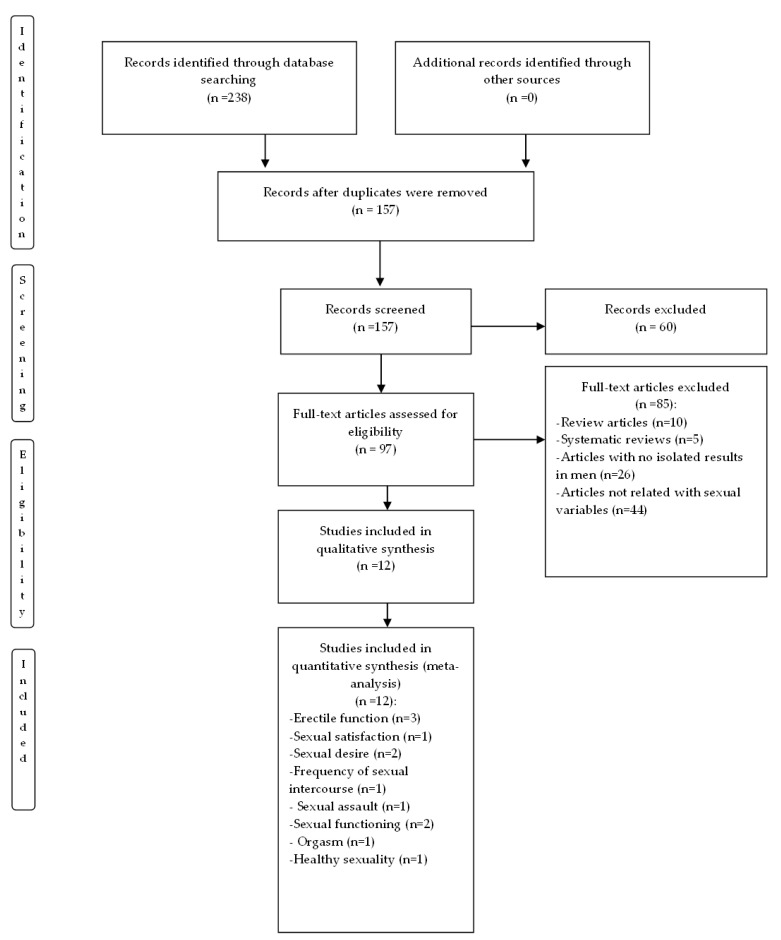
PRISMA flow in the selection of articles.

From the search strategy, a total of 238 references were identified in the electronic databases consulted, of which 157 references were reviewed after eliminating duplicates with the Zotero bibliographic reference manager. Of the remaining 157 references, 60 documents that did not meet the inclusion criteria were eliminated. In total, 97 references were selected after examining their abstracts. Another 85 articles were deleted after reading the abstracts from them, for several reasons: they were review articles, not related to sexual variables or had no isolated results in men. Finally, 12 studies that met the selected inclusion criteria were added, and the quality criteria were subjected to analysis (see Figure 1). The research papers selected are presented in Table 1. The overall quality score was 4.5 points out of 5 for the randomized controlled trials and 23 points out of 26 for the non-randomized controlled trials (see Table 2 and Table 3).

### 3.1. Randomized Control Trials

The sample of the studies included was made up of men between the ages of 14 and 71 and involved a total of 3782 men. Of these, the total number of participants in randomized clinical trials was 646 men. The study by Hucker and McCabea [44] included women. A differentiation was made between the results of men with respective assessment instruments, the International Index of Erectile Function (IIEF), and the Premature Ejaculation Diagnostic Tool (PEDT). This study showed a positive change in terms of a significant reduction in the frequency of sexual problems and distress and in sexual functioning. The study by Grensman et al. [45] was a blind randomized control trial with 94 male patients from Primary Care, in patients discharged for burnout whose sexual functioning was assessed. Research included randomized therapeutic blocks to explore whether health-related quality of life increased after a 20-week group treatment of traditional yoga (TY), mindfulness-based cognitive therapy (MBCT) [46], or cognitive-behavioural therapy (CBT). The study by Leahu and Delcea involved 500 people with premature ejaculation, randomly divided into two groups: 60-day training in various mindfulness techniques, while the control group received the same assessment instruments but no intervention. The results were mainly an improvement in the increase in the interval from the onset of erection to ejaculation as a result of the techniques learned [47].

### 3.2. Non-Randomized Control Trials

The objective of the study by Bossio et al. [36] was to determine whether it is feasible to implement an empirically supported treatment protocol tailored for 4-week female sexual dysfunction to the specific needs of men with situational erectile dysfunction. This study was applied to 10 men. The sessions lasted 2.25 h and included daily practice activities at home and integrated elements of psychoeducation, sexual therapy, and mindfulness skills. The men completed the following questionnaires: the International Index of Erectile Functioning (IIEF), the Relationship Assessment Scale (RAS), and the Five-Facet Mindfulness Questionnaire (FFMQ). This study found an improvement in sexual satisfaction and non-judgemental observation of one’s own experience. The results support the feasibility of tailoring a mindfulness-based group treatment for situational erectile dysfunction and represent a promising treatment pathway for men with this sexual dysfunction. In another study by Bossio, Higano, and Brotto [48], a 4-session mindfulness-based group intervention was applied to prostate cancer (PC) survivors and their partners. Effect sizes 6 months post-treatment indicated “moderate” improvements in overall sexual satisfaction and “large” improvements in increased mindfulness in prostate cancer survivors. Small decreases in partner-reported sexual intimacy and small increases in anxiety were also found in PC survivors and their partners.

### 3.3. Studies Describing the Impact of Mindfulness Practice on Men

These studies indicate a possible relationship between the degree of mindfulness in adolescent and adult men, with and without physical disabilities, and the level of sexual desire, sexual activity, subjective sexual arousal, greater degree of sexual satisfaction, protection against sexual insecurities, relational flourishing, sexual harmony, orgasm consistency, decreased anxiety for sexual performance, and lower rates of alcohol-related sexual assault.

The study by Déziel, Godbout, and Hébert [26] aimed to examine mindfulness as a mediator of the relationship between anxiety and sexual desire in men who consulted clinical sexology, 28.7% of them due to inhibited sexual desire. The results suggest that mindfulness can be integrated as an intervention technique in men who present anxiety and inhibited sexual desire. The study by Dosch et al. [30] explored the role of mindfulness practice in factors such as sexual desire and sexual activity (sexual satisfaction and frequency of sexual intercourse). The results revealed positive effects of conscious sexuality, such as mindfulness towards internal and external events during sexual activity, possibly improving sexual arousal and desire, as well as sexual satisfaction. For their part, Dunkley, Goldsmith, and Gorzalka [49] point out in their study that mindfulness can play a role in protecting against sexual insecurities and in improving sexual satisfaction in men.

The study by Leavitt et al. [50] concluded that awareness and non-judgement were associated with relational flourishing, sexual harmony, and consistency of orgasm. The study carried out by Gallagher, Hudepohl, and Parrott [51] provided the first support for mindfulness as a mediating factor that favours the reduction of the relationship of alcohol consumption in men with sexual coercion/aggression towards their sexual partners. The study carried out by Pereira, Teixeira, and Nobre [52] indicated a positive association between male sexual functioning and self-compassion for men with physical disabilities and a negative association for men without physical disabilities. This study is of great relevance to advance the knowledge of the practice of self-compassion and its relationship with sexuality (see Table 1).

**Table 1:** Mindfulness-based interventions in men and selected data analysis.

First Autor, Year, Country	N	Design	Procedure	Instruments	Results
Bossio et al., 2018, Canada [36]	N = 10 men with erectile dysfunction	Mixed methods: quantitative and qualitative	Psychoeducation, mindfulness exercises, and homework for 4 weeks.	International Index of Erectile Functioning (IIEF) Relationship Assessment Scale (RAS) Five-Facet Mindfulness Questionnaire (FFMQ)	Significant improvement in erectile function, general sexual satisfaction, and impartial observation of one’s own experience. Qualitative analysis revealed 6 themes: normalization, group magic, identification of effective treatment goals, increased self-efficacy, relationship factors, and treatment barriers.
Bossio et al., 2021, Canada [48]	N = 14 men with prostate cancer and their partners	Mixed methods: quantitative and qualitative	Psychoeducation, mindfulness exercises, and homework for 4 weeks for prostate cancer survivors.	Adapted Dyadic Adjustment Scale (ADAS) Global Measure of Sexual Satisfaction (GMSEX) International Index of Erectile Functioning (IIEF) Female Sexual Function Index (FSFI) Hospital Anxiety and Depression Scale (HADS) Five-Facet Mindfulness Questionnaire, short form (FFMQ-SF)	At 6-month follow-up after treatment, “moderate” improvements in overall sexual satisfaction and a significant increase in mindfulness were identified in prostate cancer survivors.
Déziel et al., 2017, Canada [26]	N = 105 men	Descriptive, correlational, quantitative, transversal	Evaluation of the variables anxiety, sexual desire, and mindfulness.	International Index of Erectile Functioning (IIEF) French Psychiatric Symptoms Index-14 (PSI-14) The Mindful Attention Awareness Scale (MAAS)	The results indicated that anxiety was related to a lower level of dispositional mindfulness, which in turn was related to a higher level of sexual desire.
Dosch et al., 2016, Switzerland [30]	N = 600 (300 men and 300 women)	Descriptive, correlational, quantitative, transversal	Evaluation of the variables sexual desire, sexual satisfaction, frequency of sexual relations, adult attachment, focus and motivation of avoidance, mindfulness and impulsivity in men who were divided into 3 groups: (a) high activity and dyadic sexual desire; (b) men with high dyadic and solo sexual activity and desire; and (c) low activity and dyadic sexual desire.	Sexual Desire Inventory (SDI) Multidimensional Sexuality Questionnaire (MSQ) Revised Experiences in Close Relationships (ECR-R) Focus-Avoidance Temperament Questionnaire (ATQ) Mindful Attention Awareness Scale (MAAS) Urgency-Premeditation-Perseverance-Sensation Seeking-Positive Urgency Impulsive Behavior Scale (UPPS-P)	The results were different for each group: participants in group (a) were the most sexually satisfied and were characterized by a balance between reward-seeking motivational tendencies and self-control skills (high approach motivation, secure attachment, high self-control, high attention); those in group (b) were moderately satisfied and showed a type of functioning predominantly characterized by impulsivity (excessively high reward motivation in females, and low self-control in men); those in group (c) were the least sexually satisfied and were characterized by high motivation to avoid negative consequences and low self-control (high avoidance motivation, insecure attachment) and a lower degree of attentiveness.
Dunkley et al., 2015, Canada [49]	N = 687 women and N = 334 men	Descriptive, correlational, quantitative, transversal	Mindfulness in protecting against a negative image of the genitals, sexual dissatisfaction, self-image, and cognitive distractions during sexual activity.	The Cognitive Distractions During Sexual Activity Scale (CDDSA) The Genital Self-Image Scale (GSIS-20) The Golombok-Rust Inventory of Sexual Satisfaction (GRISS) The Five-Facet Mindfulness Questionnaire (FFMQ)	Mindfulness accounted for a significant proportion of the variation in genital self-image. Subscales which evaluate mindfulness of the ability to describe and not make value judgements significantly predicted positive genital self-image.
Gallagher et al., 2010, USA [51]	N = 167 men	Descriptive, correlational, quantitative, longitudinal	Association between the level of mindfulness of men and the stories of alcohol consumption and sexual assault towards intimate partners.	Recommended set of six questions on alcohol use from the National Institute on Alcohol Abuse and Alcoholism (NIAAA) MAAS The Sexual Coercion Subscale of the Revised Conflict Tactics Scale (CTS-2)	Larger mean amounts of alcohol consumed and lower levels of mindfulness were associated with more frequent perpetration of sexual coercion/assault in intimate partners. The interaction effect between higher amounts of alcohol consumed and mindfulness was significant.
Grensman et al., 2018, Sweden [45]	N = 94 men	Blind randomized control trial	To assess the effects of long-term treatment (20 weeks) with traditional yoga (TY), mindfulness-based stress reduction (MBCT), and cognitive-behavioural therapy (CBT) (active control) in patients on leave for exhaustion.	The Swedish Health-Related Quality of Life Survey (SWED-QUAL)	MBCT improves sexual functioning more compared to CBT and TY.
Hucker and McCabe 2015, England [44]	N = 52 (26 women and 26 men)	Randomized control trial	To evaluate an online cognitive-behavioural therapy program for female sexual problems known as pursuing pleasure (PP) and to evaluate the effect on their male partners. Six online modules that included psychoeducation, concentration of sensations, communication exercises, cognitive exercises, and contact email of a therapist. The PP also incorporated mindfulness training and online chat groups.	International Index of Erectile Function (IIEF) Premature Ejaculation Diagnostic Tool (PEDT)	Significant before and after test improvements in erectile function, sex drive, and sexual satisfaction. However, the results of orgasmic function and PE were not significant.
Leahu & Delcea. 2022, Romania [47]	N = 500 men	Randomized control trial	All eligible participants were assessed with PEDT, IELT, and MAAS, and then randomly distributed into two groups, experimental and control. Participants in the experimental group participated in mindfulness skills training for premature ejaculation for 60 days.	Premature Ejaculation Diagnostic Tool (PEDT) Self-reported intravaginal ejaculation latency (IELT) Mindfulness Attention Awareness Scale (MAAS)	There were statistically significant differences between the experimental and control groups with respect to premature ejaculation, on the PEDT and IELT. MAAS scores correlated with improvements in IELT. In addition, 10% of them no longer met DSM diagnostic criteria for PE.
Leavitt et al., 2020, USA [53]	N = 1000 adolescent women and 1000 adolescent men	Descriptive, correlational, quantitative, transversal	To assess how mindfulness trait and mindfulness state were related to sexuality outcomes of sexually active and non-active adolescents.	Kyle Inventory of Sexual Shame (KISS) Sexual esteem and anxiety were measured using six items. Body Esteem Scale for Adolescents and Adults (BES). New General Self-Efficacy Scale (NGSES) Self-Disclosure Index (SDI) Mindful Attention Awareness Scale (MASS)	Trait mindfulness was positively related to beliefs of sexual consent, body image, self-efficacy, and sexual self-esteem, as well as lower levels of sexual shame and less sexual anxiety.
Leavitt et al., 2021, USA [50]	N = 1473 heterosexual couples	Actor–partner interdependence model within a structural equation modelling framework	To evaluate whether the two factors of sexual mindfulness, awareness and non-judgement, were linked with relational flourishing, sexual harmony, and orgasm consistency.	Sexual Mindfulness Measure (SMM) Relationship Flourishing Scale (RFS) Sexual Passion Scale (SPS) Sexual Orgasm Consistency (Qualitative inform)	Awareness was positively associated with relational flourishing, sexual harmony, and orgasm consistency in men.
Pereira et al., 2020, Portugal [52]	N = 377 participants with and without disabilities (189 women and 188 men)	Descriptive, correlational, quantitative, transversal	This study explored how mindfulness, self-compassion, and acceptance can predict sexual functioning for people with and without physical disabilities.	Five-Facet Mindfulness Questionnaire (FFMQ) Self-Compassion Scale (SCS) Acceptance and Action Questionnaire (AAQ-II) Index of Erectile Function adapted for men who have sex with men Female Sexual Function Index for women who have sex with women	Results showed that self-compassion and acceptance are significant predictors of sexual functioning for the male sample. There was a “moderate” effect of physical condition (with or without physical disabilities) on association between male sexual functioning and self-compassion. The results suggest the importance of strategies derived from third-generation therapies for sexual functioning and overall sexual health.

Note: MBCT: mindfulness-based cognitive therapy; MBSR: mindfulness-based stress reduction; CBT: cognitive-behavioural therapy; TY: traditional yoga; PE: premature ejaculation; PP: pursuing pleasure.

**Table 2:** Estabrooks scale for a non-randomized control trials.

Study	Score (/28)	Design (/6)	Recruitment (/4)	Loss Control (/4)	Description of Intervention (/6)	Statistical Analysis (/6)	Outcome Measures (/2)
Bossio et al. 2018, Canada [36]	26	6 (H)	4 (H)	2 (M)	6 (H)	6 (H)	2 (H)
Bossio et al., 2021, Canada [48]	26	6 (H)	4 (H)	2 (M)	6 (H)	6 (H)	2 (H)
Déziel et al. 2018, Canada [26]	24	6 (H)	4 (H)	0 (L)	6 (H)	6 (H)	2 (H)
Dosch et al. 2016, Switzerland [30]	28	6 (H)	4 (H)	4 (H)	6 (H)	6 (H)	2 (H)
Dunkley et al. 2015, Canada [49]	23	5 (H)	4 (H)	0 (L)	6 (H)	6 (H)	2 (H)
Gallagher et al. 2010, USA [51]	27	5 (H)	4 (H)	4 (H)	6 (H)	6 (H)	2(H)
Leavitt et al. 2020, USA [53]	27	5 (H)	4 (H)	4(H)	6(H)	6 (H)	2(H)
Leavitt et al. 2021, USA [50]	28	6 (H)	4 (H)	4(H)	6(H)	6 (H)	2(H)
Pereira et al. 2020, Portugal [52]	28	6 (H)	4 (H)	4 (H)	6 (H)	6 (H)	2 (H)

Note: H (high), M (medium), L (low).

**Table 3:** Jadad scale for a randomized control trials.

Study	Total Quality (/5)	Randomisation (/2)	Blinding (/2)	Follow-Up (/1)	Randomisation Concealment
Grensman et al. 2018, Sweden [45]	5	2	2	1	Unknown
Hucker and McCabe 2015, England [44]	4	2	1	1	Unknown
Leahu and Delcea 2022, Romania [47]	3	1	1	1	Unknown

Note: Scale adapted from Jadad et al. [42].

Non-randomized studies scored a minimum of 23/28 and a maximum of 28/28. Whether sample selection and sample losses were adequately described, whether the authors justified the sample size, whether inclusion and exclusion criteria were stated, the statistics used, and the outcome measures were assessed. Table 2 shows the scores obtained by each of the articles selected. Randomized studies are of medium to high quality. These studies are randomized and double-blinded and describe loss to follow-up.

### 3.4. Qualitative Analysis with NVIVO

Likewise, a qualitative analysis was carried out using the NVIVO version 11 program for the frequency of words in the selected articles. Since all the final articles were in English, the same language was used for the analysis.

As can be seen in the cloud of the 100 most frequent words, the ones that stand out as the most frequent were “sexual”, with 2265 words in total, followed by “mindfulness”, with 1077 words. Both words highlight the central theme referred to in the article. In order of frequency, it is followed by the word “women” (466), which curiously is more frequent than “men” (407), revealing that, despite the incipient investigation of mindfulness in male sexuality, there are more women in these studies. It is closely followed by words such as “self” (402) and “satisfaction” (332), and the latter turns out to be one of the most important elements in assessing the impact of mindfulness on sexuality. We found other frequent words, such as “functioning” (282) or “desire” (264), which refer to some of the other sexual variables that have been evaluated more frequently in the studies included in the review. It is followed by other very frequent words, such as “relationship” (221) and “partner” (212), which highlight the importance of interaction in sexuality. It is necessary to highlight some variables closely related to a change of paradigm in sexual therapy, such as “health” (202), “body” (184), or “awareness” (176).

**Figure 2:**
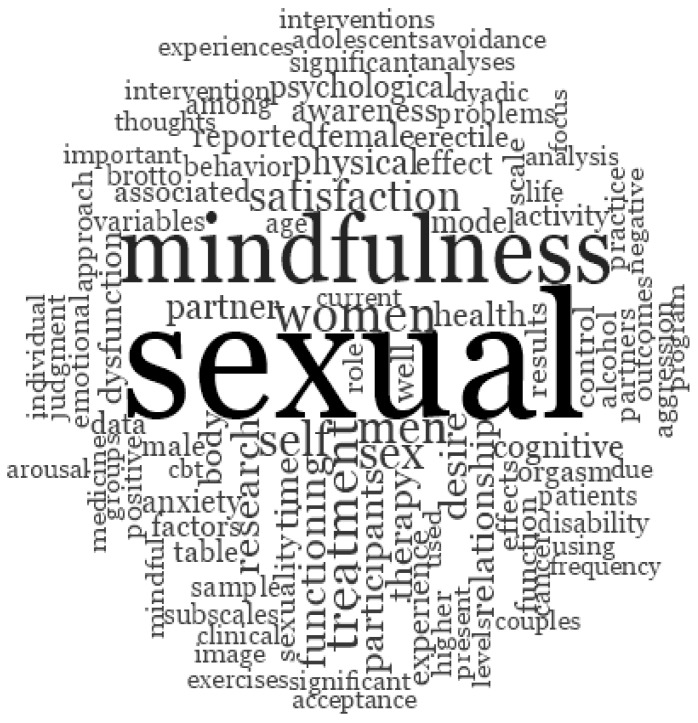
Word cloud of the selected articles in English.

On the other hand, it is worth noting the frequency of other words, such as “time” (210), “cognitive” (189), “anxiety” (176), or “control” (152), which reveal some of the factors that may influence sexual activity of men who attend therapy or variables that improve after practising mindfulness (see Figure 2).

## 4. Discussion

The review of scientific literature has revealed that there are a limited number of studies that allow a reliable evaluation of the efficacy of mindfulness-based interventions in treating male sexual problems and the impact on the experience of male sexuality. However, these studies begin to indicate that the practice of mindfulness can improve various aspects of the experience of male sexuality, for example, in sexual functioning [45,52]; erectile function [36,44]; ejaculation [47]; the degree of sexual desire [26]; arousal, marital satisfaction, and sexual satisfaction [30,48,50]; protection against sexual insecurities [40,46,49], or even partner relationships [30,50], among others.

Selected studies relate the influence of mindfulness on sexual activity. The conclusions of these studies suggest that mindfulness-based interventions could be a relevant component to be integrated in the treatment of men at different stages of their life cycle, with and without physical disabilities [52], and for males who may or may not present symptoms of anxiety or a sexual disorder [26,44,47,53]. It is worth noting that several studies highlight that the use of mindfulness and third-generation therapies could offer significant effects on men’s sexual health, e.g., [47,48,52].

The study by Hucker and McCabe [44] agrees with the results obtained by Dosch et al. [30], which explored the role of the psychological traits mindfulness, sexual desire, and sexual activity. One of the contributions of the Dosch et al. [30] study is the division into three distinct profiles related to different types of psychological functioning, which could be further explored in future studies. The adaptation of the study to men who are sexually active with other men is also recognized as an important variable to consider. Likewise, the study by Dunkley et al. [49] reveals that higher levels of mindfulness are associated with fewer sexual insecurities and greater sexual satisfaction in men. Grensman et al. [45] present a study in which an intervention with mindfulness-based cognitive therapy (MBCT) shows benefits in sexual functioning in men. These findings are related to the 2016 study by Silva, Pascoal, and Nobre [35], where they examine the mediating role of cognitive distraction in physical appearance and the relationship between beliefs about appearance and sexual functioning. In fact, as shown in the qualitative analysis of the frequency of words, cognitive control could be one of the variables that influence sexual problems due to the rebound effect generated by this strategy, which is consistent with previous studies (for example, Sánchez-Sánchez et al. [54]).

Although the objective of this review study was to explore different mindfulness-based sex therapy interventions, the results obtained indicate that there are a limited number of studies related to the evaluation of the effectiveness of mindfulness in the areas of sexuality in men. Therefore, the need arises for a rigorous investigation, isolating the therapeutic components together with an adequate sample size, where the effects of mindfulness on male sexuality are evaluated. Thus, mindfulness could improve male sexuality through a more focused attention on sexual stimuli, a better emotional connection with oneself or one’s partner, an increase in acceptance, etc. Determining the processes involved in improving male sexual health through mindfulness practice would be an important step in advancing research on this topic. Despite the limitations detected in the studies selected, based on the evidence reviewed, it seems reasonable to argue that mindfulness-based interventions represent a valuable, promising contribution, which could provide a unique way to promote sexual treatments capable of responding to the psychotherapeutic needs of men. However, to empirically establish this possibility, more process-focused randomized studies with adequate control groups are needed.

Limitations

One of the main limitations of this work is the small number of studies that have been reviewed due to the few mindfulness-based interventions on sexuality carried out in men. Likewise, another recognized limitation is the existing variability in the methodological rigour of the studies, since some lack an active control group, have a small sample size, or do not have an adequate evaluation of the different variables related to sexuality, which can generate restrictions in the knowledge of the benefits of mindfulness-based interventions in men. Likewise, another relevant limitation of this study resides in the fact that quantitative data on the effect size are not offered, which does not facilitate comparisons with other interventions carried out.

On the other hand, the samples of participants were mostly white, educated, with above-average incomes, and fairly skilled in their relationships. Future research may test whether couples with more contextual stress benefit from mindfulness training.

## 5. Conclusions

The general corollary of this scoping review refers to the effectiveness of mindfulness practice on male sexuality. Specifically, its application improves sexual satisfaction, sexual desire, erectile function, orgasmic function, and self-control. These findings may allow sex therapists and researchers to better address men’s sexual well-being and sexual satisfaction. Likewise, mindfulness practice increases variables related to self-esteem, which can be affected because of social physical standards (for example, Petisco-Rodríguez et al. [55]), and it can influence sexuality; for example, mindfulness improves body image, sexual self-esteem, and genital self-image. On the other hand, sexual shame, sexual anxiety, and the association between alcohol consumption and sexual assault decreased. These improvements also occurred in men with physical disabilities, and this represents a contribution to the population with functional diversity.

Research on the application of mindfulness to the improvement of male sexuality is still scarce, and the existing studies include women or lack methodological rigour. More randomized studies with a control group and larger samples are needed to acquire more conclusive results.

## Data Availability

The data that support the findings of this study are available from the corresponding author upon reasonable request.
